# Dysfunctional putamen modulation during bimanual finger-to-thumb movement in patients with Parkinson's disease

**DOI:** 10.3389/fnhum.2015.00516

**Published:** 2015-09-30

**Authors:** Li-rong Yan, Yi-bo Wu, Xiao-hua Zeng, Li-chen Gao

**Affiliations:** ^1^Department of Information, Wuhan General Hospital of Guangzhou CommandWuhan, China; ^2^Department of Unmanned Aerial Vehicle, Wuhan Mechanical Technology CollegeWuhan, China; ^3^Department of Radiology, Wuhan General Hospital of Guangzhou CommandWuhan, China

**Keywords:** Parkinson's disease, putamen, movement, network, functional magnetic resonance imaging

## Abstract

Parkinson's disease (PD) is a neurodegenerative disorder affecting middle-aged and elderly people. PD can be viewed as “circuit disorder,” indicating that large scale cortico-subcortical pathways were involved in its pathophysiology. The brain network in an experimental context is emerging as an important biomarker in disease diagnosis and prognosis prediction. This context-dependent network for PD and the underling functional mechanism remains unclear. In this paper, the brain network profiles in 11 PD patients without dementia were studied and compared with 12 healthy controls. The functional magnetic resonance imaging (fMRI) data were acquired when the subjects were performing a pseudorandomized unimanual or bimanual finger-to-thumb movement task. The activation was detected and the network profiles were analyzed by psychophysiological interaction (PPI) toolbox. For the controls and PD patients, the motor areas including the primary motor and premotor areas, supplementary motor area, the cerebellum and parts of the frontal, temporal and parietal gyrus were activated. The right putamen exhibited significant control > PD activation and weaker activity during the bimanual movement relative to the unimanual movement in the control group. The decreased putamen modulation on some nucleus in basal ganglia, such as putamen, thalamus and caudate, and some cortical areas, such as cingulate, parietal, angular, frontal, temporal and occipital gyrus was detected in the bimanual movement condition relative to the unimanual movement condition. Between-group PPI difference was detected in cingulate gyrus, angular gyrus and precuneus (control > PD) and inferior frontal gyrus (PD > control). The deficient putamen activation and its enhanced connectivity with the frontal gyrus could be a correlate of impaired basal ganglia inhibition and frontal gyrus compensation to maintain the task performance during the motor programs of PD patients.

## Introduction

Parkinson's disease (PD) is a common neurodegenerative disorder affecting middle-aged and elderly people. PD is related with aging, heredity, cell dysfunction and environment (Lang and Lozano, 1998a,b; Samii et al., 2004), but its pathology remains unclear. The National Institute of Mental Health suggested that, exploring the brain network profiles and dysfunction may enhance the understanding of specific bio-behavioral impairments which underpin the psychiatric disorders with complex behavioral phenotypes (Insel et al., 2010). Network dysfunction is emerging as a characteristic of the neural substrates of multiple psychiatric conditions (Friston, 1998; Schmidt et al., 2013). Studies over the past decades have demonstrated that PD can be viewed as “circuit disorder” or “network dysfunction,” indicating that multiple, large scale networks were involved in its pathophysiology (Eckert et al., 2007; Eidelberg, 2009; Göttlich et al., 2013; Zhang et al., 2015). A combined magnetoencephalographic and subthalamic local field potential recording research indicated two spatially and spectrally separated networks, i.e., a temporoparietal-brainstem network coherent with subthalamic nucleus (STN) in the alpha (7–13 Hz) band, and a predominantly frontal network coherent in the beta (15–35 Hz) band (Litvak et al., 2011). Resting-state fMRI research indicated that, PD patients at off state had significantly decreased functional connectivity in the supplementary motor area, left dorsal lateral prefrontal cortex and left putamen, and had increased functional connectivity in the left cerebellum, left primary motor cortex and left parietal cortex (Wu et al., 2009). It's believed that the dysfunction of cortico-striatal-thalamic-cortical loops leads to the motor symptoms of PD including tremor, akinesia and rigor (Lang and Lozano, 1998b; Jankovic, 2008) and the cognitive dysfunction including mild cognitive impairment (MCI; Huang et al., 2007; Lin et al., 2008; Kwak et al., 2010).

As the initial and most obvious symptoms are movement-related in the PD course, the motor function and the underlying cerebral mechanisms have become the focus of PD pathology research. The changes of the structure and functional network of basal ganglia, the abnormal oscillations of the neurons in the basal ganglia and motor-related cerebral cortex (Timmermann et al., 2003; de Solages et al., 2010), and the abnormal projection from basal ganglia to cerebral cortex (Lang and Lozano, 1998b) may relate with the motor dysfunction of PD patients. Besides basal ganglia, many literatures demonstrated that there exists abnormality in the large scale cerebral motor functional network (including cerebellum, motor cortex, frontal gyrus, etc.) of PD patients. Elevated putamen-external globus pallidus (GP) and STN-internal GP inputs), internal GPi-thalamus, caudate-putamen, and internal GPi-pedunculopontine nucleus (PPN) inputs (Asanuma et al., 2006; Eidelberg, 2009; Mure et al., 2011), and decreased metabolism in premotor cortex (PMC), supplementary motor area (SMA), and posterior parietal cortex (PPC; Asanuma et al., 2006; Ma, 2007; Eidelberg, 2009) were reported.

In recent years much attention has been devoted to characterizing the neural networks under multiple conditions (Friston et al., 1997; Friston, 1998; Yan et al., 2008) and psychophysiological interaction (PPI) analysis has become more commonly used in identifying the task-dependent functional connectivity changes (Deco et al., 2011; O'Reilly et al., 2012). PPI analysis was originally proposed by Friston et al. (1997), and promotes the understanding of the brain in terms of networks and interactions between brain regions (Bullmore and Sporns, 2009; Friston, 2011). PPI aims to identify regions whose activity is dependent on an interaction between psychological factors (the task) and physiological factors (the activity of a region of interest). Researchers have found interesting results using PPI in cognition such as conflict adaptation (Wang et al., 2015) and emotion recognition (Pulkkinen et al., 2015), and disease such as small-fiber neuropathy (Hsieh et al., 2015) and Social Anxiety Disorder (Cremers et al., 2015). PPI as the brain network in an experimental context, is emerging as an important biomarker of interest in disease diagnosis and prognosis prediction. For PD patients, this context-dependent brain network and the underling functional mechanism remains unclear.

We speculated that the dysfunctional motor network of PD patients might exhibit different profiles under different movement conditions. And considering the importance of the nuclei in the basal ganglia in PD pathology, they might play crucial roles in the context-dependent network. To test these hypotheses, a randomized unimanual or bimanual finger-to-thumb movement paradigm was designed to evaluate the impact of movement conditions on the neural networks. The motor network profiles in PD patients without dementia were investigated and compared with healthy controls using PPI analysis with the specific focus on the function of the basal ganglia. We found reduced putamen-modulation to the precuneus, cingulate gyrus, and the angular gyrus in PD patients, which implies the dysfunctional interactions and impaired basal ganglia inhibition in movements and hyperactivation/connectivity of the frontal gyrus which might be the compensation to maintain the task performance during the motor programs.

## Materials and methods

### Subjects

Fifteen PD patients were studied. Four patients were excluded because they did not follow the instruction correctly during the experiments. The remaining 11 patients ranged in age from 51 to 81 (61.5 ± 7.1) years, and included eight males and three females. The diagnosis of Parkinson's disease was based on medical history, physical and neurological examinations, response to levodopa or dopaminergic drugs, and laboratory tests and MRI scans to exclude other diseases. Patients were assessed with the Unified Parkinson's Disease Rating Scale (UPDRS; Lang and Fahn, 1989) and Mini-Mental State Examination (MMSE) while off their medications. None patient had cognitive impairments (MMSE score were ≥ 21 for the subjects with eighth grade education, ≥ 23 for the subjects with high school education and ≥ 24 for the subjects with college education). Twelve health subjects (eight males and four females) with no history of neruological, psychiatric, or medical disorders, aged from 52 to 81 (65.5 ± 10.1) years served as the control group. All the subjects were right-handed. The clinical and demographic data are shown in Table 1. Both groups were matched regarding age (*t*-test, *t* = 1.0889, *P* = 0.1443), gender (Fisher's exact test, *P* = 0.556) and MMSE score (*t*-test, *t* = 1.2194, *P* = 0.1181). All participants gave written informed consent and the study protocol was approved by the Ethics Committee of Wuhan General Hospital.

**Table 1:** **Clinical details and demographics of patients with Parkinson's disease and the normal control subjects**.

**Measure**	**Normal control subjects (*n* = 12)**	**Subjects with Parkinson's disease (*n* = 11)**
Age (years)	65.5 ± 10.1	61.5 ± 7.1
Gender, male:female	8:4	8:3
Duration of disease (years)	N/A	4.9 ± 3.9
UPDRS III score (off medication)	N/A	20.1 ± 6.3
MMSE score	27.5 ± 1.6	26.5 ± 2.3

### Experimental design and image acquisition

The subjects participated in an auditory-cueing bimanual or unimanual finger-to-thumb movement task. Three kinds of movements were elicited by an auditory instruction (move left hand, move right hand, move both hands) in a pseudo-random and balanced sequence and stopped by “stop” instruction. Each movement lasted for 8 s followed by 12 s of rest. The whole experiment lasted for 360 s. During the experiments the subjects were instructed to close their eyes and focus their attention as much as possible. Patients were scanned after their medication had been withdrawn for 4 h.

Data were acquired in a GE Signa System operating at 1.5 T with a gradient echo EPI sequence (TR = 2000 ms, TE = 40 ms, FOV = 24 cm, matrix = 64 × 64 × 24, slice thickness = 5 mm, gap = 1 mm). The 3D structural images were also acquired for each subject with the parameters TR = 12.1 ms, TE = 4.2 ms, FOV = 24 cm, matrix = 256 × 256 × 172, slice thickness = 1.8 mm and gap = 0 mm.

### Data processing

**Figure 1:**
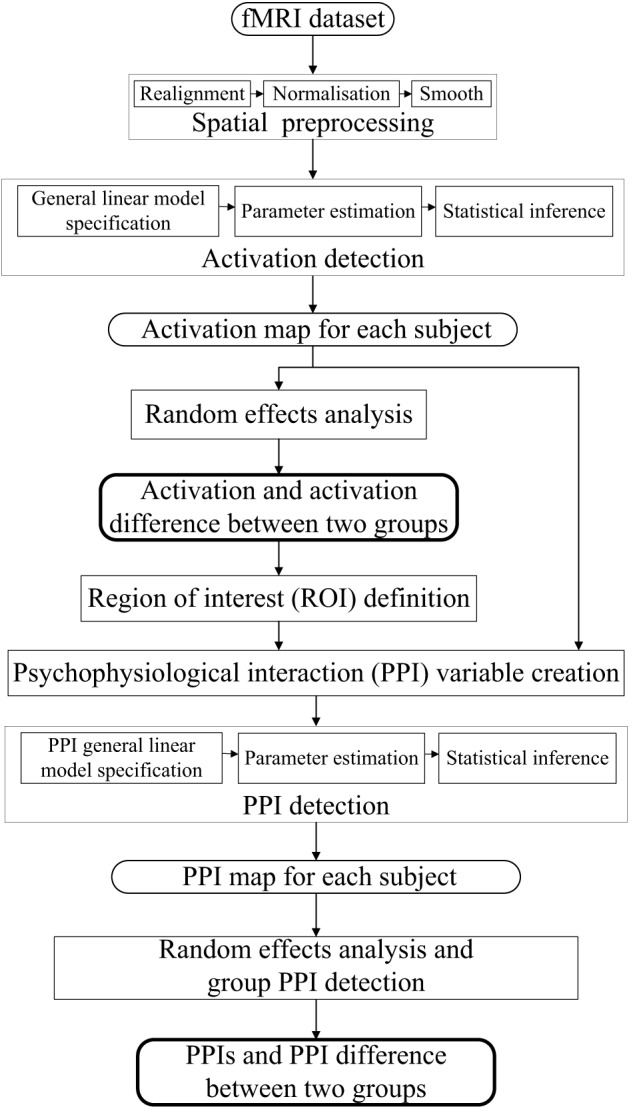
**Flow diagrams showing the processing steps for activation and PPI analysis**.

The dataset was analyzed by SPM8 software package (www.fil.ion.ucl.ac.uk/spm, Wellcome Department of Cognitive Neurology). The processing steps for the activation and PPI analysis were shown in Figure 1. The following steps were included: (i) spatial preprocessing, to make the data adequate for the analysis; (ii) activation detection, to find out the activated areas during the finger-to-thumb movement task; (iii) region of interest (ROI) definition and PPI variables extraction, to determine the specific location of the ROI and create the interaction and the main effects terms; (iv) PPI analysis, to detect the interaction between the source ROI and experimental context. In the following, the analysis procedure was elaborated.

Spatial transformation (realignment, normalization) was performed on the functional images to correct for motion and normalize to the Montreal Neurological Institute (MNI) template brain. It is noted that the magnitude (minimum to maximum) of the six realignment parameters (i.e., x, y, and z translations, pitch, roll and yaw angles) of the normal control group were (0.4216 ± 0.3240), (0.3777 ± 0.2182), (0.9240 ± 0.6210) mm, (0.6875 ± 0.4125), (0.4354 ± 0.3839), (0.5844 ± 0.4469), degrees respectively, and of the PD group were (0.3317 ± 0.2256), (0.2971 ± 0.1174), (0.9141 ± 0.5368) mm, (0.6704 ± 0.4641), (0.3380 ± 0.1547), (0.3782 ± 0.2464) degrees, respectively, which were not significantly different between two groups (*t* = 0.7652, 1.0879, 0.0407, 0.0936, 0.7839, 1.3519, *P* = 0.2263, 0.1445, 0.4840, 0.4632, 0.2209, 0.0954, respectively). The 3D structural images were utilized to determine the normalization parameters.

Three task-related regressors for left or/and right hand movement conditions were modeled as the boxcar vectors convolved with a canonical hemodynamic reference waveform. Low frequency components were removed using a high-pass filter (128 s) and the data were smoothed spatially with a Gaussian filter (full-width half-maximum (FWHM) = 8 mm). An autoregressive AR(1) model was included to account for serial correlation. The activation corresponding to each condition of each subject was detected and submitted to a second-level random effects analyses using the model of analysis of variance (ANOVA). Ages and sexes of the subjects were included as covariates. The activation of each group under three conditions were detected, and the activation difference between conditions (left + right vs. both) or groups (PD vs. control) was tested by applying appropriate linear contrasts to the ANOVA parameter estimates (*t*-test, *P* < 0.05, family-wise error (FWE) correction, extent threshold *k* > 10).

The regions with activation difference between unimanual movement and bimanual finger-to-thumb movement were defined as the ROIs. Time series from the effects of interest contrast were extracted from the ROIs, which provides an estimate of the continuous physiological response of the specific ROI (one main effect in the PPI model). The extracted time series was subsequently convolved with the contrasts of interest reflecting effects of differential movement loads, specifically, left hand movement + right hand movement > both hand movement or vice versa (the other main effect in the PPI model). The resultant interaction term was positively weighted to assess the facilitating influence of the ROI on other areas. The first level PPI maps from each subject were submitted to a second-level random effects analyses (ANOVA). The group PPIs and the PPI difference between groups were detected using *t*-test with a slightly more liberal threshold of *P* < 0.001 (uncorrected, extent threshold *k* > 10). Individual voxel peaks in significant clusters are reported in terms of MNI coordinates. The anatomical structures and the BA number were obtained using MRIcron (Rorden et al., 2007).

## Results

### Activation of two groups under three conditions

**Figure 2:**
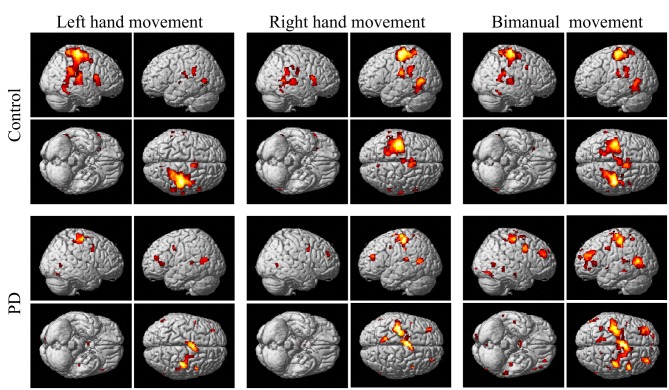
**Activation of the control and PD groups in the unimanual or bimanual finger-to-thumb movement experiments (SPM8, ***t***-test, ***P*** < 0.05, FWE-corrected, extent threshold ***k*** > 10)**. The names of the movement trials are shown in the upside. The subject groups are shown in the left side. PD, Parkinson's disease.

The group and condition specific activations are shown in Figure 2 and the details are listed in Table 2. For the controls, in the left or right hand movement conditions, the contralateral sensorimotor (BA 1, 2, 3), primary motor (M1, BA 4) and premotor (BA 6) areas, SMA (BA 6), and the ipsilateral cerebellum were activated. In addition, parts of the frontal (BA 44), temporal (BA 21, 22) and parietal gyrus and basal ganglia (putamen) were activated. In the bimanual movement condition, the bilateral motor cortical areas and cerebellum, the frontal, temporal and parietal gyrus and putamen were activated.

**Table 2:** **Anatomical structure, stereotaxic coordinates, and ***Z*** score of the activated areas in the control or patient groups**.

**Anatomical structure**	**Left hand movement**					**Right hand movement**					**Both hand movement**				
	**Peak location**			***Z* score**	**Cluster size (voxels)**	**Peak location**			***Z* score**	**Cluster size (voxels)**	**Peak location**			***Z* score**	**Cluster size (voxels)**
	***x***	***y***	***z***			***x***	***y***	***z***			***x***	***y***	***z***		
**CONTROL**															
Premotor/SMA	8	4	62	6.18	470	−26	−16	62	6.39	–	40	−26	64	7.15	1914
Primary motor/Sensorimotor	40	−26	64	7.83	5250	−38	−22	62	7.52	2517	−38	−22	62	7.08	2642
	−62	−22	20	4.96	80	−58	−22	22	5.99	380	26	−30	70	5.68	–
	–	–	–	–	–	66	−20	20	4.95	71	–	–	–	–	–
Cerebellum	−18	−54	−26	6.83	1075	14	−50	−14	6.71	872	−20	−52	−30	6.80	2102
	–	–	–	–	–	−20	−62	−24	4.86	52	14	−48	−18	6.46	–
Frontal	56	16	10	5.58	339	6	4	62	6.12	551	38	8	32	5.54	125
	46	−4	58	5.93		−2	−10	62	5.15	139	–	–	–	–	–
Temporal	−46	−64	6	5.76	124	−46	−64	6	6.79	756	−64	−40	16	5.76	267
	−48	−38	24	5.26	171	64	−30	0	5.68	728	60	−36	20	5.40	333
Parietal	–	–	–	–	–	−46	−36	26	5.75	328	−64	−24	18	5.19	116
Putamen	28	−10	8	6.65	1592	−24	4	8	6.54	721	−22	4	12	5.71	239
	−22	2	14	5.69	315	30	8	10	6.19	1000	26	12	8	5.71	758
**PD**															
Premotor/SMA	38	−26	66	5.78	717	−26	−16	60	6.11	1116	−26	−18	56	7.57	–
M1	32	−22	50	4.99	–	−36	−28	60	5.83	–	–	–	–	–	–
Cerebellum	−14	−50	−26	5.84	422	−38	−70	−22	4.51	10	22	−54	−28	5.14	253
	–	–	–	–	–	22	−60	−16	4.66	25	−18	−52	−30	5.96	–
Frontal	6	4	60	5.85	708	−6	−8	62	5.95	739	−30	38	18	6.49	1003
	−30	38	18	5.44	121	30	50	24	4.65	43	46	6	38	6.01	435
Temporal	−62	−36	8	4.68	26	−54	−56	12	5.45	179	−52	−58	8	5.97	628
	−56	−60	18	5.45	196	−52	−30	18	4.87	74	60	−60	4	5.45	47
Parietal	–	–	–	–	–	−10	−58	66	5.14	367	−10	−58	66	5.13	316
	–	–	–	–	–	–	–	–	–	–	−36	−52	56	4.84	44
Cingulate	−8	2	50	5.20	–	−8	0	50	5.36	180	−4	−42	12	6.07	2252
	−4	−42	12	5.22	203	−4	−42	12	5.52	377	–	–	–	–	–
Occipital	–	–	–	–	–	–	–	–	–	–	48	−74	−14	5.71	109
	–	–	–	–	–	–	–	–	–	–	14	−92	−8	4.85	51
Thalamus	4	−16	10	5.08	169	–	–	–	–	–	–	–	–	–	–

*For each anatomical structure, the representative regions in the left and right hemispheres are listed. The height and extent thresholds were set at P < 0.05, FWE-corrected, k > 10. The location is in MNI coordinates. PD, Parkinson's disease; SMA, supplementary motor area; M1, primary motor area*.

For the PD patients, in the left or right hand movement conditions, the contralateral primary motor (M1, BA 4) and premotor (BA 6) areas, SMA, the ipsilateral cerebellum, parts of the frontal, temporal, parietal and cingulate gyrus were activated. In the bimanual movement condition, the left premotor area and bilateral cerebellum, the frontal, temporal, parietal and occipital gyrus were activated.

### Activation difference within and between two groups

**Figure 3:**
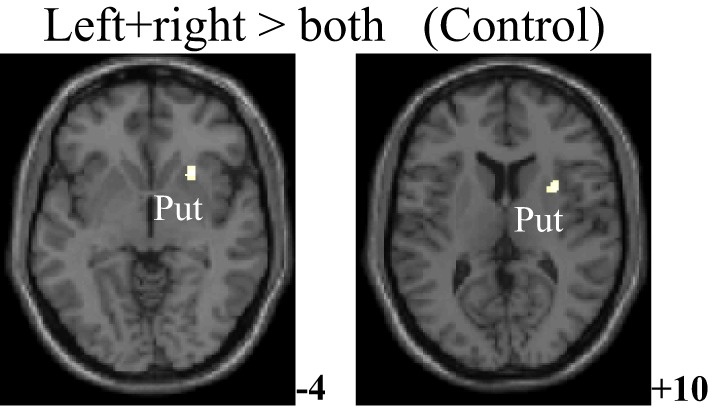
**Activation difference in the control group (left + right > both hand movement, SPM8, ANOVA, ***P*** < 0.05, FWE-corrected, extent threshold ***k*** > 10) with the peak ***Z*** score at [26, 18, −4] and [32, 8, 10]**. The images were superimposed on a standard statistical parametric mapping anatomical template brain in neurological convention with *z* coordinate in MNI space for each slice shown in the right side. Put, Putamen.

**Figure 4:**
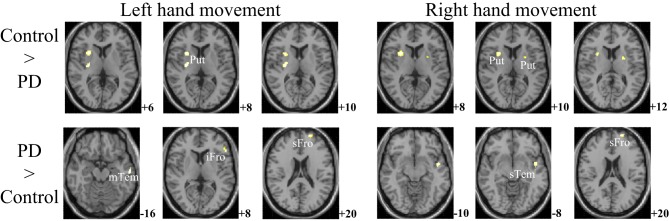
**Activation difference between control and PD groups in the left hand and/or right hand movement conditions (SPM8, ANOVA, ***P*** < 0.05, FWE-corrected, extent threshold ***k*** > 10)**. The images were superimposed on a standard statistical parametric mapping anatomical template brain in neurological convention with *z* coordinate in MNI space for each slice shown in the right side. PD, Parkinson's disease; Put, putamen; mTem, medial temporal gyrus; iFro, inferior frontal gyrus; sFro, superior frontal gyrus; mFro, medial frontal gyrus; sTem, superior temporal gyrus.

The within-group activation difference (left + right > both) was found in putamen (with peak voxel at [26, 18, −4] and [32, 8, 10]) in the control group (Figure 3) and was not found in the PD group. Activation difference between two groups under left or right handmovement conditions are shown in Figure 4. The control > PD activation mainly located in putamen in the basal ganglia. The PD > control activation mainly located in the superior frontal and temporal gyrus. There was no activation difference between two groups under the bimanual movement condition. The details of the activation difference are listed in Table 3.

**Table 3:** **Anatomical structure, stereotaxic coordinates, and ***Z*** score of the different peak areas between the activated area in the control or patient groups**.

**Anatomical structure**		**BA**	**Peak location**			***Z* score**	**Cluster size (voxels)**
			***x***	***y***	***z***		
**LEFT HAND MOVEMENT**							
Control > PD	Putamen	–	30	−12	10	3.77	81
	Putamen	–	30	12	6	3.67	116
PD > Control	Superior frontal	10	−20	62	20	4.88	61
	Inferior frontal	45	−52	30	8	4.60	48
	Middle temporal gyrus	20	−58	−14	−16	3.70	31
**RIGHT HAND MOVEMENT**							
Control > PD	Putamen	–	30	12	8	4.04	123
	Putamen	–	−24	2	12	3.38	20
PD > Control	Superior frontal	10	−20	62	20	4.34	39
	Superior temporal gyrus	48	−46	2	−10	3.73	55
**CONTROL**							
left +right > both hand movement	Putamen	–	26	18	−4	4.84	30
	Putamen	–	32	8	10	4.73	41

*The height and extent thresholds were set at P < 0.05, FWE-corrected, k > 10. The location is in MNI coordinates. PD, Parkinson's disease; BA, Brodmann's area*.

### PPIs corresponding to putamen in the two groups

**Figure 5:**
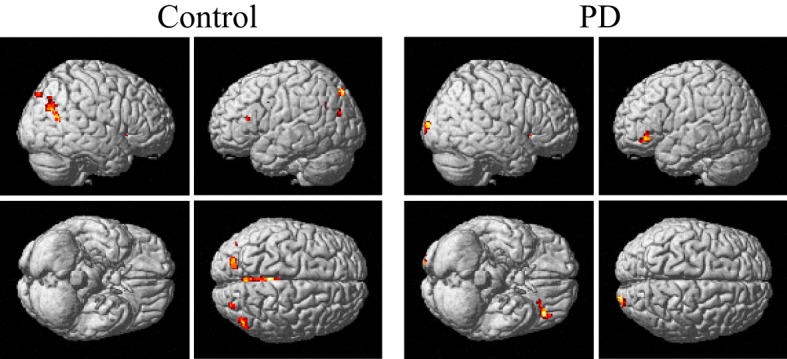
**PPIs corresponding to putamen in control and PD groups (SPM8, ANOVA, ***P*** < 0.001, uncorrected, extent threshold ***k*** > 10)**. PD, Parkinson's disease.

From Figures 3, 4 and Table 3, it can be seen that putamen might play an important role in the movement coordination of the both hands. The subject-specific PPIs corresponding to putamen (with peak voxel at [26, 18, −4] and [32, 8, 10]) were detected and then submitted to a second-level random effects analyses of variance (*P* < 0.001, uncorrected). The group-specific PPIs are shown in Figure 5 and the details are listed in Table 4. For the controls, decreased modulation of putamen on cingulate, parietal, frontal, occipital, angular and temporal gyrus, putamen and thalamus were detected in the bimanual movement condition relative to the unimanual movement condition. While for PD patients, the decreased modulation of putamen were detected in frontal and occipital gyrus, extra-nuclear, thalamus, putamen and caudate.

**Table 4:** **Anatomical structure, stereotaxic coordinates, and ***Z*** score of the peak areas in the PPI profiles in the control or patient groups**.

**Anatomical structure**	**BA**	**Peak location**			***Z* score**	**Cluster size (voxels)**
		***x***	***y***	***z***		
**CONTROL**						
Cingulate	23	10	−32	34	4.55	1447
Precuneus	7	−4	−62	36	4.10	–
Superior parietal	19	−22	−84	48	4.04	84
Precentral	44	−40	2	28	3.77	52
Putamen	−	22	14	2	3.67	42
Inferior frontal	45	−46	28	16	3.55	12
Superior occipital	19	28	−84	42	3.51	31
Angular	39	−36	−62	30	3.47	23
Angular	39	44	−64	30	3.40	179
Middle temporal	37	54	−60	16	3.32	–
Thalamus	–	−14	−14	4	3.29	11
**PD**						
Putamen	–	26	18	−4	4.45	21
Caudate	–	−12	14	4	3.97	73
Inferior frontal	47	−40	36	−8	3.80	98
Superior occipital	17	24	−98	10	3.79	63
Caudate	–	18	10	8	3.75	148
Putamen	–	28	2	6	3.71	33
Thalamus	–	−12	−18	8	3.30	10

*The height and extent thresholds were set at P < 0.001, uncorrected, k > 10. The location is in MNI coordinates. PD, Parkinson's disease; BA, Brodmann's area*.

### PPI difference between two groups

**Figure 6:**
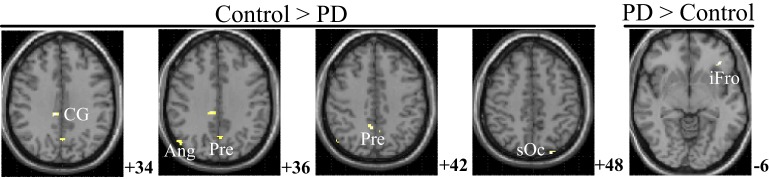
**PPI difference between control and PD groups (SPM8, ANOVA, ***P*** < 0.001, uncorrected, extent threshold ***k*** > 10)**. The images were superimposed on a standard statistical parametric mapping anatomical template brain in neurological convention with *z* coordinate in MNI space for each slice shown in the right side. PD, Parkinson's disease; CG, Cingulate gyrus; Ang, Angular gyrus; Pre, Precuneus; sOc, superior occipital gyrus; iFro, inferior frontal gyrus.

Figure 6 and Table 5 depict the difference of PPIs corresponding to putamen between control and PD groups (*P* < 0.001, uncorrected), which included cingulate gyrus, angular gyrus, superior occipital gyrus and precuneus (control > PD) and inferior frontal gyrus (PD > control).

**Table 5:** **Anatomical structure, stereotaxic coordinates, and ***Z*** score of the different peak areas between the PPI profiles in the control or patient groups**.

**Anatomical structure**		**BA**	**Peak location**			***Z* score**	**Cluster size (voxels)**
			***x***	***y***	***z***		
Control > PD	Angular	39	52	−70	38	3.84	26
	Superior occipital	7	−24	−84	48	3.82	10
	Cingulate	23	10	−32	34	3.71	27
	Precuneus	7	8	−50	42	3.50	20
	Precuneus	7	0	−64	36	3.18	19
PD > Control	Inferior frontal	47	−38	32	−6	3.45	22

*The height and extent thresholds were set at P < 0.001, uncorrected, k > 10. The location is in MNI coordinates. PD, Parkinson's disease; BA, Brodmann's area*.

## Discussion

### General characteristics of the activation and network profiles of control and PD groups

For the controls and PD patients, the motor areas including the primary motor and premotor areas, SMA, and the cerebellum were activated in the movement conditions. These motor areas play the important roles in processing sensory information, and planning or executing hand movement (Moritz et al., 2000; Umests et al., 2002). Besides these areas, parts of the frontal, temporal and parietal gyrus were activated in both groups. As for the activation difference between two groups, generally the sizes of the activated areas, especially the motor cortex, were larger in the control group than those in the PD group, which was in accordance with the former results (Wu et al., 2010). The putamen was strongly and bimanually activated in control group, but not activated in the PD group in the movement conditions (Figure 2, Table 2). The right putamen exhibited significant control > PD activation difference (Figure 4, Table 3). In addition, the right putamen seemed to have weaker activity during the bimanual movement relative to the unimanual movement in the control group (Figure 3, Table 3). All these results implicated the importance of putamen, especially the right putamen, in hand movement and the coordination of two hands and its dysfunction in PD. The activation of the left frontal and temporal gyrus was stronger apparently in PD group than that in control group (Figure 4, Table 3).

The control of the hands involves a distributed network in which interactive processes task place between many neural assemblies to ensure efferent organization and sensory integration (Wu et al., 2010). Hence exploration on the interaction among brain regions may be more important than simply detecting the activation areas in understanding the coordination of the two hands. In consideration of the important finding of putamen with significant within-group and between-group activation difference and our hypothesis on the crucial role of the basal ganglia in the context-dependent network, the PPIs corresponding to putamen were explored. For the controls, decreased modulation of putamen on cingulate, parietal, frontal, occipital, angular and temporal gyrus, precuneus, putamen, and thalamus were detected in the bimanual movement condition relative to the unimanual movement condition. While for PD patients, the decreased modulation of putamen were detected in frontal and occipital gyrus, thalamus, putamen, and caudate (Figure 5, Table 4). For both groups, the PPIs corresponding to putamen included not only some nucleus in basal ganglia, such as putamen, thalamus and caudate, but also some cortical areas, such as frontal and occipital gyrus. Between group PPI difference was detected in cingulate gyrus, angular gyrus, and precuneus (control > PD) and inferior frontal gyrus (PD > control). See Figure 6, Table 5. Generally the PPI scope in the control group was much larger. Several studies reported the relatively reduced functional connectivity in PD patients (van Eimeren et al., 2009; Skidmore et al., 2011; Hacker et al., 2012). Specifically in accordance with our results, the functional connectivity corresponding to putamen seemed weaker in PD patients (Hacker et al., 2012). Lower striatal correlations with thalamus, midbrain, pons and cerebellum in PD patients (Hacker et al., 2012), decreased activity in the putamen and increased cortical activity in the frontal lobe (Disbrow et al., 2013) have been reported. In our study, the PPI map of the control group was generally symmetric except in the putamen and thalamus, while the PPI map of the PD group was obviously asymmetric, which was similar with former observation in the functional connectivity of PD patients (Barnes et al., 2010; Hacker et al., 2012).

### Key regions in the activation and network profiles of control and PD groups

In the activation and network profiles of the control and PD groups, two regions, i.e., the putamen and frontal regions seemed to play the specific roles. The putamen exhibited the control > PD activation and left + right > both hand movement activation within the control group. The frontal gyrus exhibited PD > control activation and connectivity with the putamen. These two regions are specifically crucial in the cortico-subcortical network and frontal network of the PD patients (Litvak et al., 2011).

The human brain network of motor function is composed of basal ganglia, cerebral motor cortex and cerebellum, among which the basal ganglia connect dorsal thalamus, ventromedial nucleus, premotor area and prefrontal cortex, and play the critical role in the complex cortical-subcortical circuits, i.e., the basal ganglia-thalamus-cortex circuits (Alexander and Moeller, 1994). Many researchers have emphasized the pathophysiology of PD as degeneration of dopaminergic nigrostriatal neurons with consequent dysfunction of these circuits (Lang and Lozano, 1998b; Jankovic, 2008; Hacker et al., 2012). The basal ganglia serve motor control functions such as scaling or focusing of movements (Alexander and Crutcher, 1990), and sustain the balance between facilitation and suppression of movements (Mink, 1996). Previous finding on functional connectivity indicated that the dorsal striatum (caudate and putamen) preferentially receives inputs from motor, sensory and premotor cortices, the ventral striatum (the nucleus accumbens and the olfactory tubercle) receives afferent inputs from cingulate cortex (Graybiel et al., 1994; Brooks, 1995). Specifically the putamen is the projection site of the cortical inputs into the basal ganglia and its activity is mainly movement related instead of cognition related (Kraft et al., 2007). Histologically, afferent fibers from the dorsal part of the putamen project somatotopically to the lateral parts of the substantia nigra (SN), which relates to the motor circuit system, and fibers from the caudate project to the rostral nigra, which relates to cingulate and association cortical system (Parent and Hazrati, 1994). In accordance with these results, we observed the putamen modulation on the thalamus, cingulate gyrus and the association area in control group (Table 4). The putamen activity and its modulation to cingulate gyrus and the association area seemed larger in control group than in PD group (Tables 3, 5).

The functional role of the basal ganglia in bimanual coordination isn't quite clear till now. Putaminal activity was the greatest during the period of motor task initiation and was critical in the neural control of bimanual coordination (Kraft et al., 2007). An animal experiment found that the majority of the 58 recorded neurons in the basal ganglia exhibited a significant modulation of activity in unimanualtrials irrespective of the movement hand and one-third of the neurons exhibited activity reflecting a bimanual synergy, suggesting a possible role for basal ganglia in bimanual co-ordination (Wannier et al., 2002). In PD patients, the disturbed effective connectivity between prefrontal cortex, premotor areas, and putamen were reported (Ceballos-Baumann et al., 1994; Rowe et al., 2002; Wu et al., 2010). As the important node in the direct and indirect efferent pathways in the basal ganglia, the striatum can influence the basal ganglia output and exert either excitatory or inhibitory effect on the movement behavior including action selection as well as execution (Disbrow et al., 2013; Freeze et al., 2013). In PD, the abnormal interconnection among putamen, SN and GP causes excessive inhibition of the thalamus, and results tremors and difficulty in voluntary movements of the patients (DeLong and Wichmann, 2007). Because the putamen had stronger activation in the unimanual movement than bimanual movement in the control group, we speculated that the putamen mainly had the inhibitory effect and its function should be weakened to include more areas in bimanual movement. The control > PD putamen activation is reasonable considering that the weakened putamen inhibition would result in more involuntary movement in PD patients.

The activation of the left superior frontal gyrus and the connectivity between the left inferior frontal gyrus and putamen was stronger in PD group than that in the control group. These results implied the abnormal function of the left frontal gyrus in PD patients. The activity of the frontal area is related with the shift of the attention and the executive control, which are the crucial pre-movement processes (Wu et al., 2010; Disbrow et al., 2013). There is evidence indicating the hemodynamic responses in the mesiofrontal and sensorimotor cortex, putamen/pallidum, thalamus, and cerebellum and the participation of frontal area in the network for motor preparation (Riecker et al., 2005), movement initiation (Toxopeus et al., 2012), amplitude adjustment (Fabbri et al., 2012; Davare et al., 2015), and speed adjustment (Michely et al., 2015). It is noted that the executive functions of the PD patients may be affected even at early stages of the disease which would result in impaired motor planning, response preparation, and inhibition (Obeso et al., 2011; Toxopeus et al., 2012; Michely et al., 2015). While we noted that in our research, the patients reported no obvious difficulty in performing the unimanual or bimanual finger-to-thumb movements and the preserved movement performance was observed. The hyperactivation/connectivity of the prefrontal cortex was suggested to constitute a compensatory mechanism in the patients' hypodopaminergic state to maintain the task performance at normal levels (Rowe et al., 2002; Wu et al., 2010; Michely et al., 2015). We spectulated that the stronger activation and connectivity with putamen of the left frontal gyrus may express the compensatory neuroplasticity during the motor programs.

### Limitations and conclusion

The present study is limited principally by the relatively small size of the subject samples. This limitation is partially because of the rigorous quality assurance standards applied to the fMRI data. However, our results need to be replicated in much larger size of samples. Furthermore, because of the small sample size, the patient group can't be subdivided according to their motor symptoms and phenotypes, which have been demonstrated to functionally relate with the brain activities (Rajput et al., 2008; Bunzeck et al., 2013). Further studies might help in establishing which pathological features are common and different between motor phenotypes.

In contrast to cortical and cerebellar activity, the activity of the basal ganglia is more inconsistently reported in fMRI motor studies (Lehéricy et al., 2006), which may be caused by small signal change in the subareas (for example about 0.5% in the putamen; Lehéricy et al., 2006), the movement paradigm (externally or internally generated), the imaging resolution inefficiency to discriminate the substructures (GP vs. putamen) of basal ganglia (Scholz et al., 2000). It's possible that SN degeneration is common to all the PD patients, but the factors in other parts of the basal ganglia distinguish between phenotypes (Rajput et al., 2008; Bunzeck et al., 2013). Several studies have indicated that there existed different grades of connectivity depending on the striatal subdivision, such as posterior putamen > anterior putamen connectivity with the brainstem (Hacker et al., 2012). In our research, the dysfunction of the putamen was observed, but the further exploration within this region was not performed. In addition, when detecting PPI and PPI difference, we applied the uncorrected hypothesis test. These limitations are subject to the current research condition that only the 1.5T MRI scanner with the relatively low signal-to-noise ratio (SNR) is available in our hospital, which has the inefficient spatial resolution and can only generate the signal with small changes in these substructures of the basal ganglia. MRI scanner with higher magnetic field strength or the multi-modal neuroimaging is promising to solve these issues.

There exists the formal possibility that the drug discontinuance time (4 h) might be not long enough to eliminate the medication effects. We didn't request the subjects to withdraw the drug for 1 day or to take their usual medication like other research (Hacker et al., 2012) based on the consideration to fulfill a tradeoff between the impact of head motion on imaging and the impact of medication on data analysis. This warrants the further research to include both the medicated and unmedicated states. The fMRI studies of the unmedicated (drug-naive) patients are challenging and some powerful regression techniques might help to reduce the motion-related artifacts (Helmich et al., 2010). Further studies might examine how PPI differences change depending on medication status and types.

In this paper, the brain network profiles in 11 PD patients without dementia were studied and compared with 12 healthy controls. The right putamen exhibited significant control > PD activation difference and weaker activity during the bimanual movement relative to the unimanual movement in control groups. The PPIs corresponding to the putamen (with peak voxel at [26, 18, −4] and [32, 8, 10]) were explored. Between group PPI difference was detected in cingulate gyrus, angular gyrus and precuneus (control > PD) and inferior frontal gyrus (PD > control). PD patients exhibited reduced putamen activation as well as modulation to the cingulate gyrus, angular gyrus, and precuneus during the finger-to-thumb movement, which implies the impaired basal ganglia inhibition in movements. The hyperactivation/connectivity of the frontal gyrus is the compensation to maintain the task performance during the motor programs.

### Conflict of interest statement

The authors declare that the research was conducted in the absence of any commercial or financial relationships that could be construed as a potential conflict of interest.
